# Research on the Histological Features and Pathological Types of Gastric Adenocarcinoma With Mucinous Differentiation

**DOI:** 10.3389/fmed.2022.829702

**Published:** 2022-03-04

**Authors:** Nian-Long Meng, Yang-kun Wang, Hai-Li Wang, Jun-Ling Zhou, Su-nan Wang

**Affiliations:** ^1^Department of Pathology, 989th Hospital of the Joint Logistic Support Force of the PLA, Luoyang, China; ^2^Department of Pathology, Foresea Life Insurance Guangzhou General Hospital, Guangzhou, China; ^3^Shenzhen Nanshan District People's Hospital, Shenzhen, China; ^4^Shenzhen Polytechnic, Shenzhen, China

**Keywords:** gastric neoplasm, mucinous adenocarcinoma, mucinous differentiation, histopathology, immunohistochemistry

## Abstract

**Objective:**

To discuss the histological features, pathological types, and prognosis of gastric adenocarcinoma with mucinous differentiation.

**Methods:**

Specimens of 189 cases of gastric adenocarcinoma with mucinous differentiation were collected for detailed histomorphology, immunohistochemistry, fluorescence *in situ* hybridization, and follow-up.

**Results:**

In accordance with the morphological and histological structural features of the cancer cells as well as the area ratio of the mucus, gastric adenocarcinoma with mucinous differentiation was divided into four types, namely pure mucinous carcinoma, intraductal papillary mucinous carcinoma, signet ring cell type mucinous carcinoma, and mixed cell type mucinous carcinoma. Based on the macroscopic types according to Bormann's classification, pure mucinous carcinoma was mostly Type I, intraductal papillary mucinous carcinoma was mostly Type II, signet ring cell type mucinous carcinoma was mostly Type IV, and mixed cell type mucinous carcinoma was mostly Type III. The 5-year survival rate was 69.2, 64.2, 0, and 31.5%, respectively. There was a statistical difference in the lymph node metastasis rate and survival rate of the four carcinoma types. The invasion features of pure mucinous carcinoma entailed penetrating corrosively in a push-in form, without blood vessel or lymphatic metastasis and with few lymphocytes and lymphatic nodules in the marginal area. Thus, there was little lymph node metastasis and invasion of nerves. The HER2 protein expression rate was 40.2% (76/189), the HER2 gene amplification detected by FISH technology was 16.9% (32/189).

**Conclusion:**

The independent histological type, four subtypes, and histopathological classification of gastric mucinous adenocarcinoma are important for the prognosis evaluation and precise treatment of this disease.

## Introduction

A gastric neoplasm is a highly heterogeneous tumor, as reflected throughout its occurrence, recurrence, and metastasis, from the tumor's histological morphology, immunophenotype, and DNA ploidy to its molecular biology and genetics (1–4). As early as 1965, Laurén put forward a classification of gastric cancer into an intestinal type, diffuse type, and mixed type (5). A classification of mixed gastric mucinous adenocarcinoma (6, 7) was proposed in 2010 and 2019 in *WHO Classification of Tumours of the Digestive System* by the World Health Organization (WHO). At present, the WHO and Laurén classifications are mostly used in clinicopathological diagnoses. There are multiple histological types of mixed adenocarcinoma of the stomach, including a mix of differentiated and undifferentiated types, a mix of neuroendocrine carcinoma and gastric adenocarcinoma, and a mix of gastric mucinous adenocarcinoma and differentiated/undifferentiated adenocarcinoma (8, 9). Gastric mucinous adenocarcinoma is a type of common gastric cancer, with a WHO International Classification of Disease for Oncology code of 8,480/3 (10–12). It is usually accompanied by tubular adenocarcinoma, papillary adenocarcinoma, or signet ring cell carcinoma tissues. The prognosis of mucinous carcinoma is poor, especially with widely differentiated signet ring cells (13). The HER2 gene amplification and protein expression of mixed gastric mucinous adenocarcinoma, key points of determination, joint detection, and significance of HER2 gene amplification markers, and sensitivity of chemotherapy drugs have been proposed in previous studies (14–16). In this study, 189 cases of gastric adenocarcinoma with mucinous differentiation have been collected for further research on the morphological features, histopathological type, HER2 gene amplification, and prognosis of the disease to provide a reliable pathological basis for precise clinical treatment.

## Materials and Methods

### Demographics

The data of a total of 189 patients with a pathological diagnosis of gastric adenocarcinoma with mucinous differentiation in the 989th Hospital of the Chinese People's Liberation Army Joint Logistic Support Force, Shenzhen Nanshan Hospital, from September 2011 to September 2016 were collected. The patients ranged from 35 to 92 years old, with an average age of 58.7.

### Methods

After the operation, all specimens were fixed within 30 min with 10% neutral buffered formalin that was freshly prepared for 8–48 h, with a fixative solution-to-tissue volume ratio of 10:1. The tissues in tumor areas were fully and conventionally cut according to the depth of invasion, color, and texture. If the tumor diameter was <3 cm, the tumor, including its peripheral area, needed to be cut out completely. For a gastric neoplasm with a diameter ≥ 4 cm, 10~15 slices were cut from each case, and no < four slices were cut from the boundary of the tumor and normal gastric tissues. The dimensions of the cut tissue areas were 2 × 1.5 × 0.3 cm. All tissue blocks were included in the proportional division of tumor morphology. In addition, one slice was cut from both the proximal and distal resection margin tissues. Two slices were cut from the deepest invasion point and the nearest serosal layer. All lymph nodes and cancer nodules were cut in different areas. Then, hematoxylin-eosin (H&E) staining, light microscopy observation, immunohistochemistry, and gene detection were conducted.

### Macroscopic Type

According to the specific gastric cancer classification proposed by (17) that is now widely used worldwide, the morphology of the tumor on the mucosal surface, and the tumor invasion mode in the gastric wall, the collected tumor specimens were classified as Type I (nodular mushroom type), Type II (local ulcer type), Type III (rodent ulcer type), and Type IV (diffuse invasion type).

### Histological Type

In accordance with the histological classification of gastric cancer (7) and pathology of gastric neoplasms (18) in *WHO Classification of Tumours of the Digestive System* (2019), gastric adenocarcinoma with mucinous differentiation was divided into four types according to the area ratio of the mucus and morphological and histological structural features of the cancer cells scattered in the mucus: pure mucinous carcinoma, intraductal papillary mucinous carcinoma, signet ring cell type mucinous carcinoma, and mixed cell type mucinous carcinoma.

### Immunohistochemistry Staining

The EnVision^TM^ two-step method was used for the staining. The tissue sections were deparaffinized, hydrated, and washed with distilled water. The sections were kept in Tris Buffered Saline (TBS) for 10 min. Endogenous peroxidase was blocked for 5 min, and then, the sections were treated with TBS for 10 min. The sections were incubated with each antibody (CKpan, CEA, villin, CDX2, HER2, p53, and Ki-67) at room temperature for 30 min. After being washed in TBS for 10 min, the sections were incubated in EnVision^TM^. After being washed in TBS for 10 min once again, the sections were incubated with the secondary antibody for 10 min. The chromogenic substrate solution was incubated for 10 min, rinsed with distilled water, stained with DAB, and counterstained with hematoxylin. Known sections of gastric mucosa were used as positive controls, while phosphate buffer saline buffer instead of the primary antibody was used as negative controls. The working solutions were purchased from Shenzhen Dartmon Biotechnology Co., Ltd., and the operation procedures were performed in strict accordance with the kits' instructions.

### HER2 Determination

Positive results were determined by the cell membrane expression. If the cell membrane was not stained, the result was 0; if the membrane of the tumor cell was poorly or indistinctly stained, the result was 1+. If the basilar, lateral, or intact membrane of the tumor cell was faintly to moderately stained, the result was 2+. If the basilar, lateral, or intact membrane of the tumor cell was positively and strongly stained, the result was 3+. Regarding the determination of the positive staining area, if the tumor cell membrane was not stained, it was negative. If ≥80% of the area was stained, it was the generalized type. If 21~79% of the area was stained, it was the partial type. If ≤ 20% of the area was stained, it was the focal type. The sections were read by two pathologists blinded to other information (14–16).

### FISH Detection

#### Reagent, Probe, and FISH Operation Steps

The Paraffin Pretreatment Kit II (mainly containing pretreatment and protease solutions) and Path VysionTM HER-2 Probe Kit were purchased from Vysis Corporation. The pretreatment procedure and FISH operation steps for the paraffin-embedded gastric cancer tissue sections followed the references (14–16) and the instructions provided for the kits.

#### Determination of FISH Results

The HER2-positive areas of the sections were detected by immunohistochemistry staining *via* the FISH technique. First, the positive area of the gastric adenocarcinoma cells was identified on an H&E-stained section. The same visual field was found on the FISH section under a 10 × objective lens, and the whole section was observed under a 40 × objective lens. If the nuclei of more than 75% of the cancer cells had hybridization signals, the results were satisfactory. At least 30 cancer cells that had intact boundaries and were isolated and non-overlapped were counted under a 100 × objective lens.

Evaluation criteria of HER2 gene amplification: If the HER2/CEP17 ratio was ≥2.0 and the mean HER2 copy number/cell was ≥4.0, it was judged as FISH positive. If the HER2/CEP17 ratio was <2.0 and the mean HER2 copy number/cell was <4.0, it was judged as FISH negative. If the HER2/CEP17 ratio was <2.0 and the mean HER2 copy number/cell was ≥4.0 and <6.0, signals in at least 20 nuclei needed to be recounted. If the results were changed, the two results had to be comprehensively judged and analyzed. The HER2/CEP17 ratio of the group was <2.0 and the mean HER2 copy number/cell was ≥6.0, so it was judged as FISH positive (high polysomic cells for short). If numerous HER2 signals were linked in clusters, it was directly judged as FISH positive. In the group, it was classified into cluster amplification, large granular amplification, and dot amplification.

### Follow-Up

The follow-up, which was conducted over the phone or *via* a letter sent to the patients or their families, ended on September 30, 2021.

### Statistical Analysis

All data were analyzed by the SPSS 22.0 software. The correlation between the expressions of HER2 in the gastric cancer tissues and the clinicopathological features was analyzed by an χ2 test. The correlation between the two methods was analyzed by a Pearson Correlation Coefficient method. A *P-*value < 0.05 was statistically significant.

## Results

### Clinical Features

A total of 189 cases of gastric adenocarcinoma with mucinous differentiation were collected. The patients comprised 112 males and 77 females, with a male-to-female ratio of 1.65:1. For all histological types, there were more male than female patients. The patients whose onset age was ≤ 60 accounted for 62.4% of cases (118/189), and the patients whose onset age was >60 accounted for 37.6% (77/189) (see Table 1).

**Table 1:** Onset age of gastric adenocarcinoma with mucinous differentiation.

**Histological type**	**Cases**	**Gender**	**Year**	
	**(%)**	**Male(%), Female(%)**	**≤60(%)**	**>60(%)**
Pure mucinous carcinoma	13(6.9)	7(53.8), 6(46.2)	7(53.8)	6(46.2)
Intraductal papillary mucinous carcinoma	95(50.3)	58(61.1), 37(38.9)	62(65.3)	33(34.7)
Signet-ring cell type mucinous carcinoma	27(14.3)	16(59.3), 11(40.7)	16(59.3)	11(40.7)
Mixed cell type mucinous carcinoma	54(28.6)	31(57.4), 23(42.6)	33(61.1)	21(38.9)
	189	112(59.3), 77(40.7)	118(62.4)	71(37.6)

### Macroscopic Type

Pure mucinous carcinoma: the nodular mushroom type was the main type, accounting for 22.2% of cases (42/189). Intraductal papillary mucinous carcinoma: the local ulcer type was the main type, accounting for 30.2% of cases (57/189). Signet ring cell type mucinous carcinoma: the diffuse invasion type was the main type, accounting for 16.9% of cases (32/189). Mixed cell type mucinous carcinoma: the ulcer type was the main type, accounting for 30.7% of cases (58/189) (see Table 2).

**Table 2:** Comparison for macroscopic type, histological stages, and lymph node metastasis of gastric adenocarcinoma with mucinous differentiation.

**Histological type**	**Cases**	**Borrmann type(%)**	**Histological stages(%)**	**Lymph node Metastasis(%)**	
	**(%)**	**I, II, III, IV**	**T1/T2, T3/T4**	**Yes**	**No**
Pure mucinous carcinoma	13(6.9)	9(69.2), 4(30.8), 0(0.0), 0(0.0),	9(69.2), 2(15.4)	0(0.0)	13(100.0)
Intraductal papillary mucinous carcinoma	95(50.3)	33(34.7), 53(55.8), 9(9.5), 0(0.0),	58(61.1), 37(38.9)	63(66.3)	32(33.7)
Signet-ring cell type mucinous carcinoma	27(14.3)	0(0.0), 0(0.0), 6(22.2), 21(77.8),	4(14.8), 23(85.1)	23(85.2)	4(14.8)
Mixed cell type mucinous carcinoma	54(28.6)	0(0.0), 0(0.0), 17(31.5), 37(68.5),	22(40.8), 32(59.3)	26(48.1)	28(51.9)
	189	42(22.2), 57(30.2), 32(16.9), 58(30.7),	93(49.2), 94(49.7)	112(59.3)	77(40.7)

### Histological Features and Pathological Type

**Figure 1:**
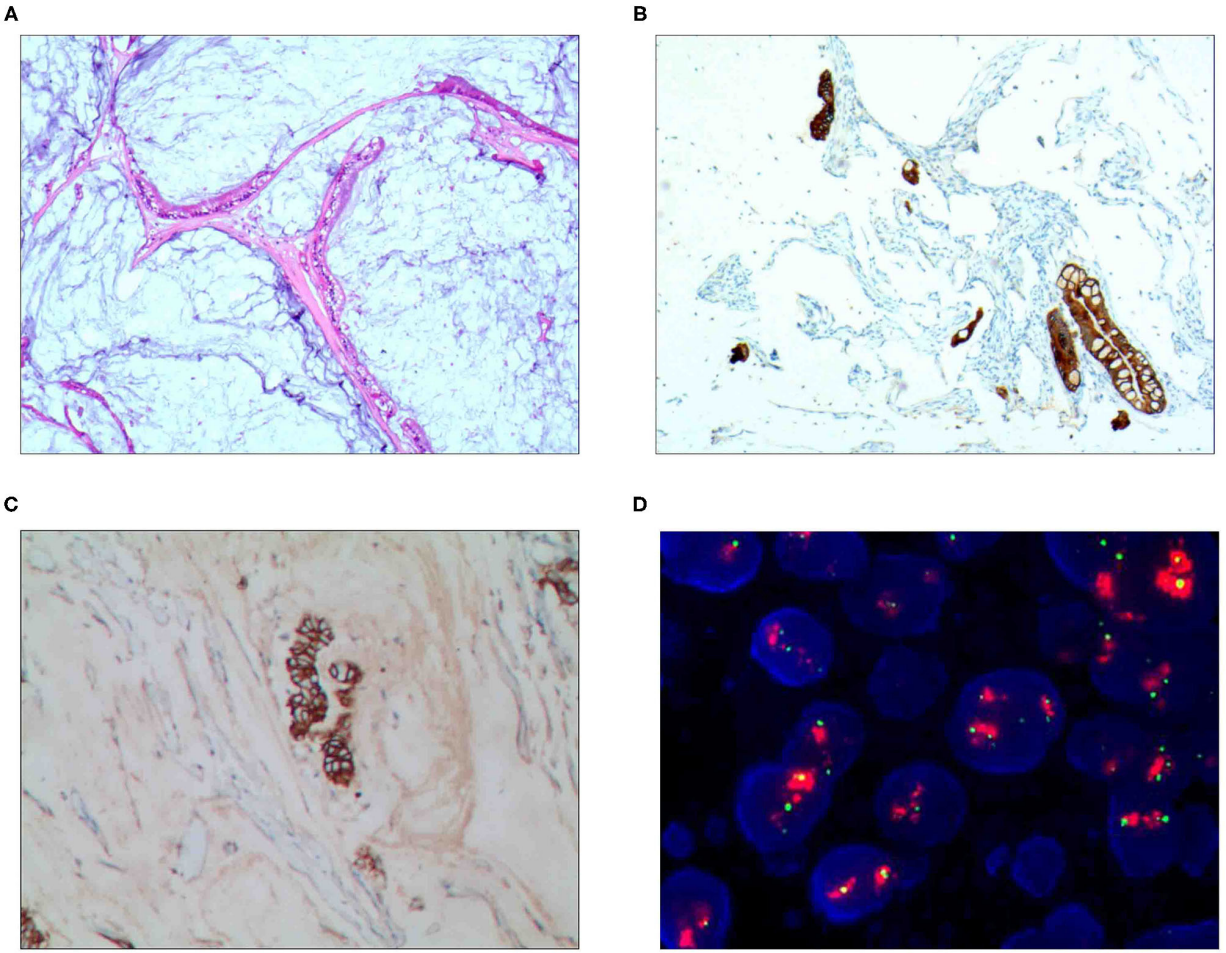
Pure mucinous carcinoma. **(A)** It was composed of differentiated adenocarcinoma cells and mucinous tissues, wherein mucus was the main tissue, accounting for more than 90%; H&E staining, × 100. **(B)** CKpan was positively expressed in the differentiated adenocarcinoma cells; EnVision™ method, × 100. **(C)** The HER2 protein was positively expressed in the differentiated adenocarcinoma cells; EnVision method™, × 100. **(D)** The HER2 gene was amplified in a cluster shape; red stands for the probe signal, and green stands for chromosome 17. The FISH method was used here.

**Figure 2:**
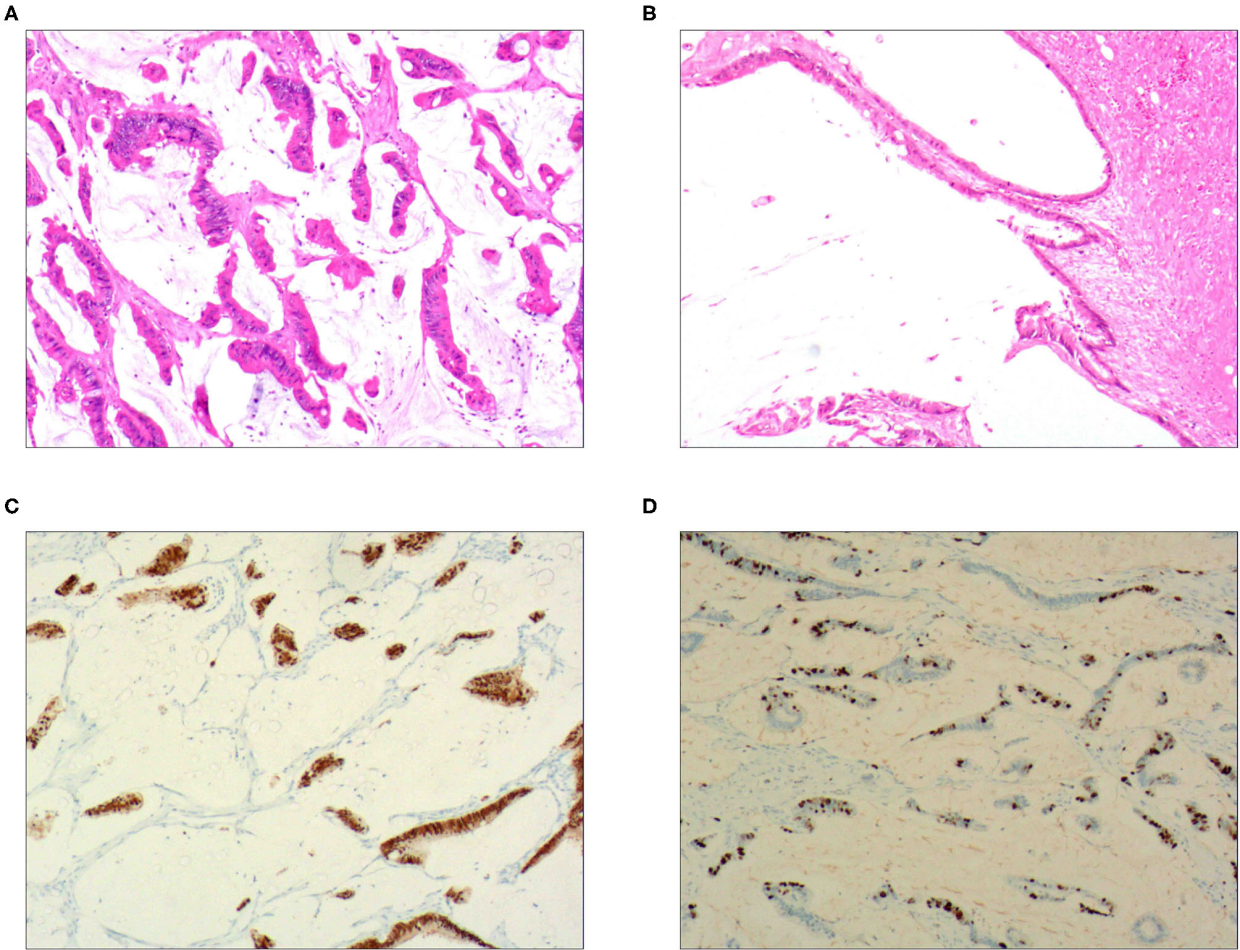
Intraductal papillary mucinous carcinoma. **(A)** It was composed of differentiated tubular and papillary adenocarcinoma tissues and mucinous tissues; H&E staining, × 100. **(B)** The tumor's primary invasion feature was to penetrate the periphery with propulsion type; H&E staining, × 100. **(C)** CDX2 was positively expressed in the differentiated adenocarcinoma cells; EnVision™ method, × 100. **(D)**. 50% of cells were positive for cell proliferation index Ki-67; EnVision™ method, × 100.

**Figure 3:**
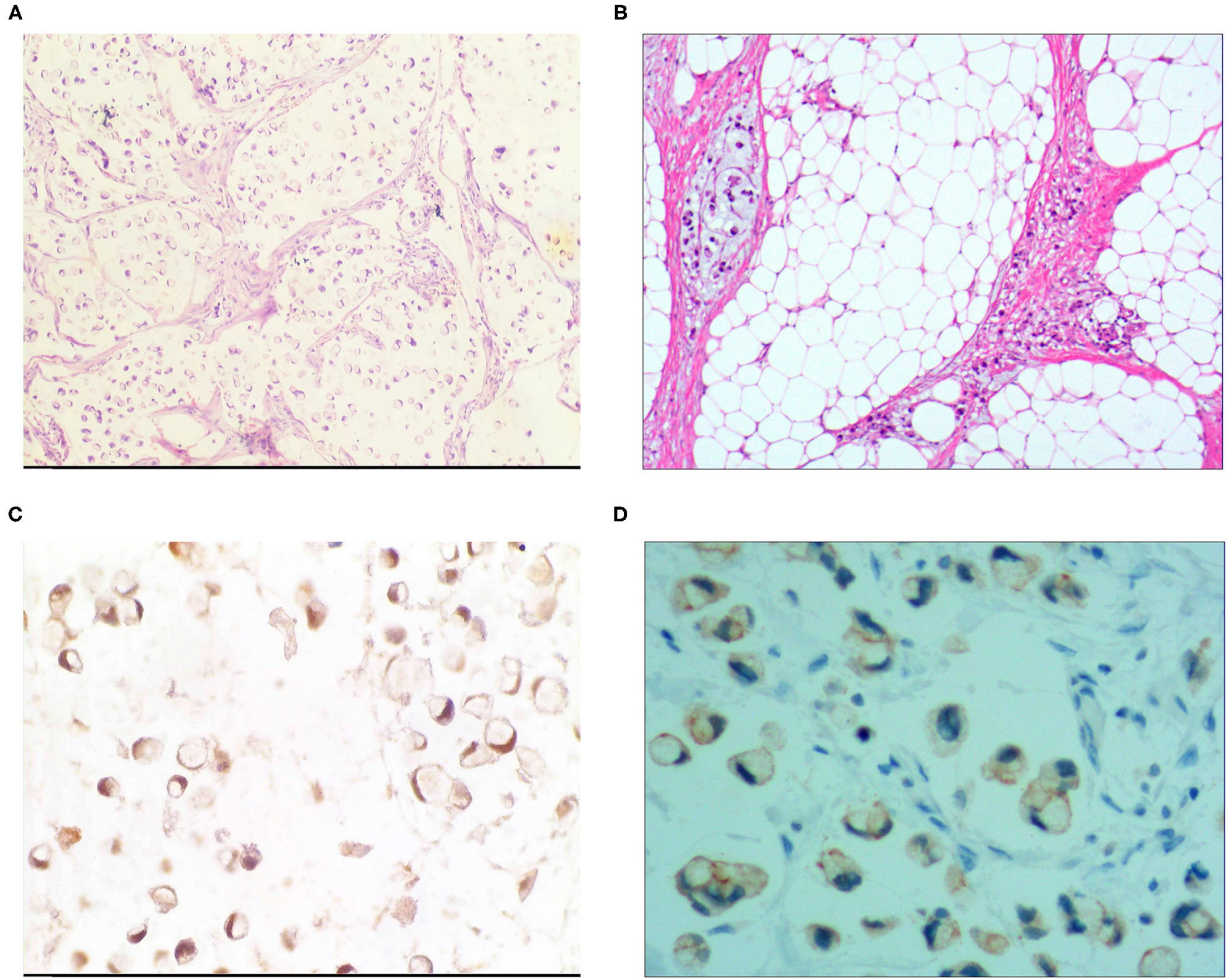
Signet ring cell type mucinous carcinoma. **(A)** It was composed of signet ring cell carcinoma cells and mucinous tissues. The cells accounted for 20-80% of the tumor; H&E, × 100. **(B)** The tumor's primary invasion feature was to grow diffusely, jumpily, and invasively and then invade the spaces among the surrounding tissues, lymphatic vessels, and blood vessels to destroy the tissues; H&E, × 100. **(C)** CKpan was positively expressed in the cancer cells; EnVision method™, × 400. **(D)** DX2 was positively expressed; EnVision method™, × 400.

**Figure 4:**
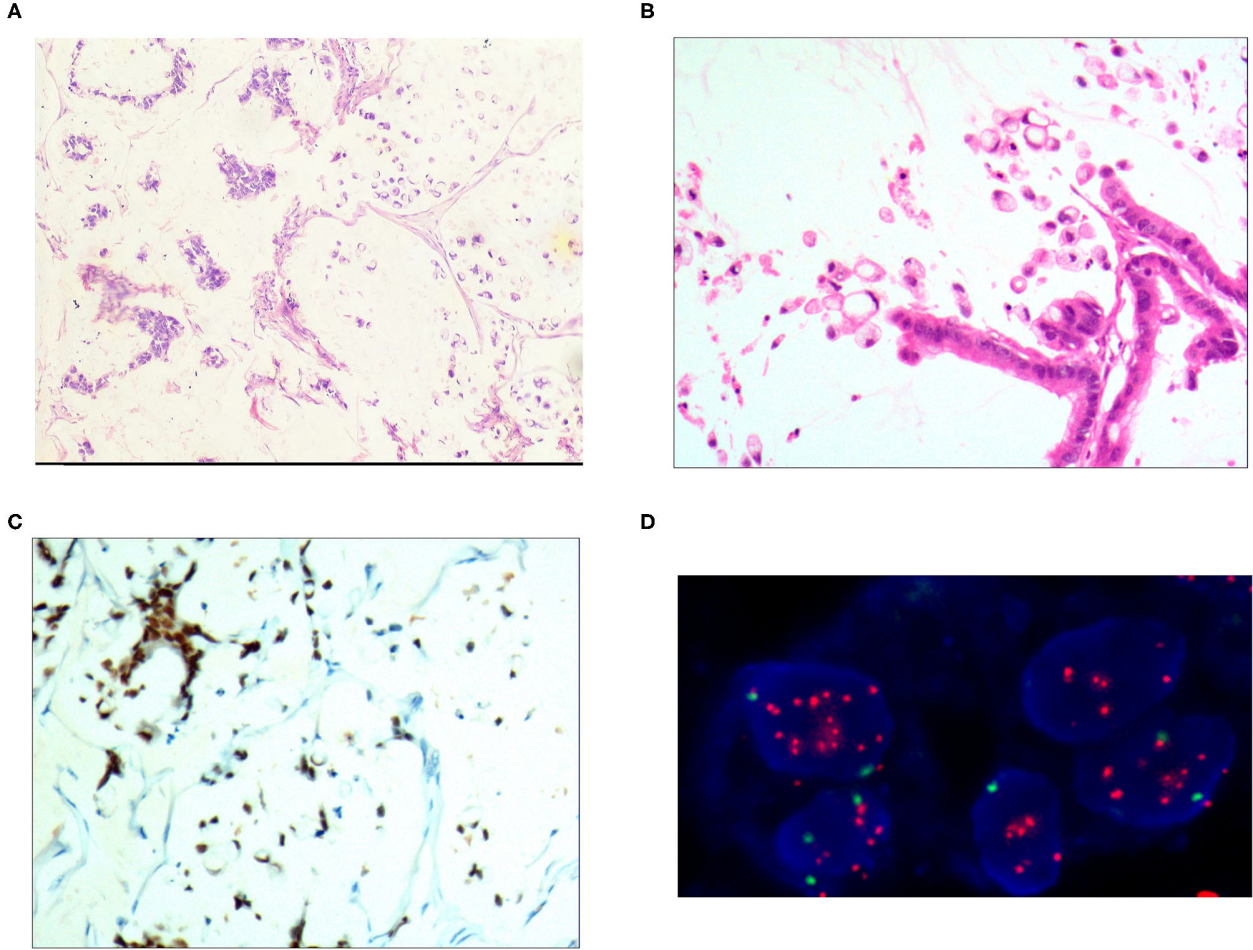
Mixed cell type mucinous carcinoma. **(A)** It was composed of cells of differentiated tubular and papillary adenocarcinoma and signet ring cell carcinoma and large areas of mucinous tissues; H&E, × 100. **(B)** The differentiated adenocarcinoma had a columnar shape, and the signet ring cell carcinoma cells had a typical signet ring structure; H&E, × 200. **(C)** CDX2 was positively expressed in the differentiated adenocarcinoma and signet ring cell carcinoma cells; EnVision™ method, × 100. **(D)** The HER2 gene was amplified in a dot shape; red stands for the probe signal, and green stands for chromosome 17. The FISH method was used here.

For the pure mucinous carcinoma, histologically, a large area of mucinous tissues was divided into irregular slices or cubes of different sizes by fibrous connective tissue, so it was also called a lake of mucus (Figure 1A). The intraductal papillary mucinous carcinoma was composed of differentiated tubular and papillary adenocarcinoma tissues and mucinous tissues (Figure 2A). The tumor's primary invasion feature was to penetrate the periphery with propulsion type (Figure 2B). The signet ring cell type mucinous carcinoma was composed of signet ring cell carcinoma cells and mucinous tissues (Figure 3A). The tumor's primary invasion feature was to grow diffusely, jumpily, and invasively and then invade the spaces among the surrounding tissues, lymphatic vessels, and blood vessels (similar to how tree roots grow into soil) to destroy the tissues (Figure 3B). The mixed cell type mucinous carcinoma was composed of differentiated tubular and papillary adenocarcinoma tissues and cells of undifferentiated signet ring cell type carcinoma (Figure 4A). The differentiated adenocarcinoma had a high columnar shape, and the cells of the signet ring cell type carcinoma had a typical signet ring structure (Figure 4B). See Table 3 for the clinicopathological diagnostic criteria of the various types of gastric adenocarcinoma with mucinous differentiation.

**Table 3:** Clinicopathologic features of gastric adenocarcinoma with mucinous differentiation.

**Histological type**	**Macroscopic type**	**Histological features**	**Immunopheno type**
Pure mucinous carcinoma	Nodular mushroom type was the main	Histologically, tumor is composed of differentiated adenocarcinoma cells and mucus tissues. Mucus is the main tissue, accounting for more than 90%, and cancer cell tissues are <10% of tumor tissues Differentiated adenocarcinoma cells are scattered among mucus tissues in nest shape and small adenoid structures. Mucus and differentiated cancer cells are divided into irregular slices or cubes of different sizes by a few fibrous connective tissues. The invasion feature of the tumor is to invade the periphery corrosively and penetratively in a push form, instead of spaces among the surrounding tissues, lymphatic vessels and blood vessels. During invasion, a large number of fibrous tissues are first produced, and then a large number of mucous tissues and a small number of adenocarcinoma cells are pushed forward to the periphery. Reactive lymphoid hyperplasia around the cancer is rare; and cancerous lymph node metastasis around the cancer is even more.	CKpan, CEA, villin, CDX2, p53; Ki67 positive cells 20-40%
Intraductal papillary mucinous carcinoma	Local ulcer type is the main	Histologically, tumor is composed of differentiated tubular and papillary adenocarcinoma tissues, and mucus tissues Differentiated tubular and papillary adenocarcinoma tissues account for 20-80% of the tumor. Differentiated adenocarcinoma cells are scattered among mucus tissues in nest shape and small adenoid structures. Mucus and differentiated cancer cells are divided into irregular slices or cubes of different sizes by fibrous connective tissues. The invasion feature of the tumor is to invade the periphery penetratively in a push form. During invasion, some fibrous tissues are pushed forward to the periphery together with mucous tissues and adenocarcinoma cells. Reactive lymphoid hyperplasia around the cancer is common, and cancerous lymph node metastasis around the cancer is common. The reactive lymphoid hyperplasia around the cancer and lymph nodes with metastatic cancer account for 50% respectively.	CKpan, CEA, villin, CDX2, p53; Ki67 positive cells 30~80%
Signet-ring cell type mucinous carcinoma	Diffuse invasion type was the main	Histologically, tumor is composed of signet-ring cell carcinoma tissues and mucus tissues. Cells of signet-ring cell carcinoma account for 20-80% of the tumor. Signet-ring cancer cells are distributed in mucus tissues singly or diffusely. Mucus and cells of signet-ring cell carcinoma are divided into irregular slices or cubes of different sizes by fibrous connective tissues. The invasion feature of the tumor is to grow diffusely, jumpily and invasively, then invade the spaces among the surrounding tissues, lymphatic vessels and blood vessels as tree roots grow into soil, and then invade and destroy the surrounding tissues. The lymph node metastasis rate is high, and the enlarged cancerous lymph node metastasis rate accounts for more than 80%, and 100% of individual cases.	CKpan, CEA, villin, CDX2, p53; Ki67 positive cells 40~60%
Mixed cell type mucinous carcinoma	Diffuse invasion type was the main	Histologically, the tumor is composed of such cells and mucinous tissues as differentiated tubular and papillary adenocarcinoma tissues, and undifferentiated cancer cells, and cells of signet-ring cell carcinoma. Differentiated and undifferentiated cancer cells account for 20-80%. Cancer cells and mucus tissues are divided into irregular slices or cubes of different sizes by fibrous connective tissue. The invasion feature of the tumor is to grow diffusely, jumpily and invasively, then invade the spaces among the surrounding tissues, lymphatic vessels and blood vessels, and then invade and destroy the surrounding tissues. Reactive lymphoid hyperplasia around the cancer and cancerometastasis is high, and the enlarged cancerous lymph node metastasis is higher.	CKpan, CEA, villin, CDX2, p53; Ki67 positive cells 40~80%

### Immunophenotype

For the pure mucinous carcinoma scattered cancer cells, CKpan (Figure 1B), CEA, villin, CDX2, and p53 were positively expressed; 20~40% of cells were positive for cell proliferation index Ki-67. For the intraductal papillary mucinous carcinoma cells, CKpan, CEA, villin, CDX2 (Figure 2C), and p53 were positively expressed; 30~80% of cells were positive for cell proliferation index Ki-67 (Figure 2D). For the signet ring cell type mucinous carcinoma cells, CKpan (Figure 3C), CEA, CDX2 (Figure 3D), villin (Figure 4C), and p53 were positively expressed; 40~60% of cells were positive for cell proliferation index Ki-67 (Figure 2C). For the mixed cell type mucinous carcinoma cells, CKpan, CEA, villin, CDX2, and p53 were positively expressed; 40~80% of cells were positive for cell proliferation index Ki-67.

### Relationship Between HER2 Gene Amplification and Protein Expression Rate

The HER2 protein expression localization was on the cell membrane, and the positive expression rate was 40.2% (76/189). Here, HER2 protein expression 3+ accounted for 16.4% of cases (31/189), including 2 cases of the 3+ generalized type, 11 cases of the partial type (Figure 1C), and 18 cases of the localized type (Figure 2C). Moreover, HER2 protein expression 2+ accounted for 13.8% of cases (26/189), including 1 cases of the 2+ generalized type, 2 cases of the partial type, and 17 case of the localized type. The HER2 protein expression 1+ accounted for 10.1% of cases (19/189), including 0 cases of the 1+ generalized type, 6 cases of the partial type, and 13 cases of the localized type. A total of 113 cases were negative, accounting for 59.8% (113/189). The HER2 gene amplification rate detected by FISH was 16.9% (32/189), including 6 cases of HER2 gene cluster amplification, 11 cases of large granular amplification (Figure 1D), and 10 cases of dot amplification (Figure 4D), and 5 cases of high polysomy. See Table 4 for the relationship between the HER2 gene protein expression and HER2 gene amplification of gastric cancer.

**Table 4:** Comparison of HER2 gene protein expression and HER2 gene amplification in tissues of 189 cases of gastric adenocarcinoma with mucinous differentiation.

**Group**	**HER2 protein expression rate (%)**	**HER2 gene amplification (%)**
-	113/189(59.8)	0
+	19/189(10.1)	0
++	26/189(13.8)	1/26(3.8)
+++	31/189(16.4)	31/31(100.0)
Total	76/189(40.2)	32/189(16.9)

### Follow-Up Results

The patients with a survival rate <1 year accounted for 78.3% of cases (148/189), those with a survival rate of 2~4 years accounted for 62.4% (118/189), and those with a survival rate of >5 years accounted for 46.0% (87/189) (see Table 5).

**Table 5:** Comparison of survival rate of gastric adenocarcinoma with mucinous differentiation.

**Histological type**	**Cases**	**Survival Rate (** ***n*** **,%)**		
	**(%)**	**<1 year**	**2-4 years**	**>5 years**
Pure mucinous carcinoma	13(6.9)	13(100.0)	12(83.3)	9(69.2)
Intraductal papillary mucinous carcinoma	95(50.3)	82(86.3)	74(77.9)	61(64.2)
Signet-ring cell type mucinous carcinoma	27(14.3)	18(66.7)	9(33.3)	0
Mixed cell type mucinous carcinoma	54(28.6)	35(64.8)	23(42.6)	17(31.5)
	189	148(78.3)	118(62.4)	87(46.0)

## Discussion

The histopathological types of gastric cancer mainly include well-differentiated tubular adenocarcinoma, moderately differentiated tubular adenocarcinoma, poorly differentiated adenocarcinoma, signet ring cell carcinoma, mucinous adenocarcinoma, and papillary adenocarcinoma (19). The clinicopathological features of gastric mucinous adenocarcinoma remain unclear, and the pathological diagnostic criteria are not yet unified. The main problems are that the histological type, amount of mucus, and area ratio of mucus of gastric mucinous adenocarcinoma cells are difficult to establish and that integrative molecular spectra and putative therapeutic targets have not been established (20, 21). It has been reported that the survival outcomes of gastric mucinous adenocarcinoma are poor (11). It has also been stated that the prognosis of gastric mucinous adenocarcinoma is good, especially that of Stage III gastric mucinous adenocarcinoma (22). In recent years, several scholars have considered the mixed alterations of mucinous carcinoma and signet ring cell carcinoma to be a unique histopathological type and classified it as mucinous adenocarcinoma with signet ring cells due to the different onset ages and invasion features of lymphatic vessels (13, 23). In this study, gastric mucinous adenocarcinoma with signet ring cell carcinoma was more common in young adults, especially women, with an average age of 32.1 years. The main macroscopic type was the diffuse invasion type, with signet ring cancer cells and lakes of mucus tissue. The invasion of lymph cells around the cancer was mostly positive. The number of reactive lymphoid hyperplasias was small, but the amount of cancerous lymph node metastasis obviously increased. The 5-year survival rate was zero, and 69.2% of patients died within 1 year. Gastric mucinous adenocarcinoma (except for signet ring cell carcinoma) was accompanied by tubular and papillary adenocarcinoma, with an average onset age of 56.3. The main macroscopic type was the rodent ulcer type, which was histologically divided into differentiated adenocarcinoma, undifferentiated carcinoma, and signet ring cell carcinoma, with lakes of mucus tissue. The invasion of lymph cells around the cancer was mostly positive. The number of reactive lymphoid hyperplasias increased, and the 5-year survival rate was 11.1%. Therefore, the names signet ring cell type mucinous carcinoma and mixed cell type mucinous carcinoma were proposed in the study.

Here, the mucinous adenocarcinoma had rich extracellular mucin, and abnormal development and a unique molecular background manifested clinically and pathologically. Common molecular and genetic changes of the mucinous colorectal adenocarcinoma were determined according to the current study results and the references used (24). The mucinous adenocarcinoma showed high microsatellite instability. The RAS/RAF/MAPK and PI3K/AKT pathways showed different mutation rates, but the common mucinous pathways could not be determined (25). In this study, in accordance with the observation and follow-up of the 189 cases of gastric mucinous adenocarcinoma, if mucus tissue accounted for more than 90% and cancer cell tissue accounted for <10% of the tumor tissue, the cancer was named gastric mucinous adenocarcinoma or pure type mucinous carcinoma, and its unique clinicopathologic features were determined. Pure mucinous carcinoma was common in younger patients, and the average age of onset in this study was 39.6. The main macroscopic type was the nodular mushroom type, the histological structural features of which are as follows: (1) Mucus produced from the epithelium reaches the mesenchyme to form interstitial mucous bursa. The interstitial mucous bursa has no epithelial cells, while the cellular mucous bursa has epithelial cancer cells. (2) Both the interstitial mucous bursa and epithelial mucous bursa are invasive. Regarding the invasion features, the tumor penetrates jumpily and corrosively in a push-in form, without blood vessel or lymphatic metastasis and with few lymphocytes and lymphatic nodules in the marginal area. The invasion of lymph cells around the cancer is mostly negative, the number of local reactive lymphoid hyperplasias is small, and lymph node metastasis either cannot occur or rarely occurs. Therefore, recurrent cases can be treated by excision several times, and the 5-year survival rate is 69.2%.

In accordance with the clinicopathological features, computerized tomography scan features, and molecular and genetic features of the gastric mucinous adenocarcinoma, the RAS/RAF/MAPK and PI3K/AKT pathways showed different mutation rates (26–28). The prognosis of gastric mucinous adenocarcinoma varies greatly based on different histopathological diagnostic criteria (26–28). The diagnosis is affected by the different tissues and proportions of cells of the adenocarcinoma. The extracellular mucin of gastric mucinous adenocarcinoma accounts for at least 50% of the tumor volume (24). Based on the histomorphology and follow-up of the 189 cases of gastric adenocarcinoma with mucinous differentiation covered in this study, the prognosis differed when the quantity of mucus accompanying non-mucinous carcinoma exceeded 10% or the non-mucinous carcinoma tissues accompanying mucinous carcinoma exceeded 10%. According to the nature and quantity of the mucus in gastric mucinous adenocarcinoma and the different accompanying cancer cells, the grade malignancy varied greatly. The disease manifestations could be divided into pure mucinous carcinoma, intraductal papillary mucinous carcinoma, signet ring cell type mucinous carcinoma, and mixed cell type mucinous carcinoma according to the malignancy grade (from low to high). The patient age of onset, macroscopic type, histological features, lymphocyte reaction, degree of cancerous lymph node metastasis, and survival rate of the four carcinoma types differed. The average onset age of the mixed cell type mucinous carcinoma was 56.3 years. The main macroscopic type was the rodent ulcer type, which was histologically divided into differentiated adenocarcinoma, undifferentiated carcinoma, and signet ring cell carcinoma, with lakes of mucus tissue. The invasion of lymph cells around the cancer was mostly positive, the number of reactive lymphoid hyperplasias increased, and the 5-year survival rate was 11.1%. The results showed that the grade malignancy of pure mucinous carcinoma was low and that mucus of mucinous adenocarcinoma can be considered a degenerative change. Pure mucinous carcinoma has been considered (12) an indolent adenocarcinoma with a good prognosis.

In general, HER2 gene status determines the effect of targeted therapy for gastric adenocarcinoma. Therefore, accurate HER2 detection is extremely important for patients who choose targeted therapy. At present, HER2 gene detection for gastric adenocarcinoma has become a regular procedure in pathology departments, and HER2 detection guidelines have been formulated in many countries (29). The detection of HER2 for gastric cancer is different from that for breast cancer. It has extensive morphological heterogeneity, and the results are affected by multiple factors, such as laboratory quality control and determination of the staining results. For gastric carcinoma tissue, the HER2 protein expression is 6.8~34.0% (30–32), differing greatly from the positive expression rate, and the main factor is the strong heterogeneity of gastric cancer. In this study, the intensity and scope of HER2 detection were emphasized. HER2 protein detection was divided into generalized, partial, and localized types. HER2 gene detection can be divided into cluster amplification, large granular amplification, dot amplification and high polysomy, which is helpful for clinicians to master the detection results, guide medication and judge prognosis.

To sum up, in this study, gastric adenocarcinoma with mucinous differentiation was divided into four types according to the cancer cell morphology, histological structural features, and amount and area ratio of mucus: pure mucinous carcinoma, intraductal papillary mucinous carcinoma, signet ring cell type mucinous carcinoma, and mixed cell type mucinous carcinoma. The 5-year survival rate was 69.2% (9/13), 64.2% (61/95), 0% (0/27), and 31.5% (17/54), respectively. The invasion features of pure mucinous carcinoma entailed penetrating corrosively in a push-in form, without blood vessel or lymphatic metastasis and with few lymphocytes and lymphatic nodules in the marginal area. Thus, there was little lymph node metastasis and invasion of nerves. The prognosis differs significantly depending on the amount of mucus and histological type of cancer cells. The independent histological type, four subtypes, and histopathological classification of gastric mucinous adenocarcinoma are important to the prognosis evaluation and precise treatment of the disease. The pathogenesis of gastric adenocarcinoma with mucinous differentiation needs to be observed in a large number of cases and further studied from a molecular biology viewpoint.

## Data Availability Statement

The original contributions presented in the study are included in the article/supplementary material, further inquiries can be directed to the corresponding author/s.

## Ethics Statement

The studies involving human participants were reviewed and approved by Ethics Committee of The 989th hospital of PLA joint logistics support force. The patients/participants provided their written informed consent to participate in this study.

## Author Contributions

Y-kW, N-LM, and S-nW: conception and design of the research. J-LZ: acquisition of data. S-nW: analysis and interpretation of the data. H-LW: statistical analysis. Y-kW: obtaining financing. Y-kW and N-LM: writing of the manuscript. S-nW and Y-kW: critical revision of the manuscript for intellectual content. All authors read and approved the final draft.

## Funding

This work was supported by Key Scientific and Technological Tackling Plan Projects of Henan Province (132102310008).

## Conflict of Interest

The authors declare that the research was conducted in the absence of any commercial or financial relationships that could be construed as a potential conflict of interest.

## Publisher's Note

All claims expressed in this article are solely those of the authors and do not necessarily represent those of their affiliated organizations, or those of the publisher, the editors and the reviewers. Any product that may be evaluated in this article, or claim that may be made by its manufacturer, is not guaranteed or endorsed by the publisher.
